# O/W microemulsion droplets diffuse through hydrogel network to achieve enhanced transdermal drug delivery

**DOI:** 10.1080/10717544.2021.1983073

**Published:** 2021-10-01

**Authors:** Lina Shen, Xiaolin Hou, Zhi Wang, Teng Guo, Zehui He, Shuyao Ruan, Zhenda Liu, Hang Ruan, Yongtai Zhang, Nianping Feng

**Affiliations:** a Department of Pharmaceutical Sciences, Shanghai University of Traditional Chinese Medicine, Shanghai, China; b Department of Pharmacy, The People’s Hospital of Hebi, Hebi, China

**Keywords:** Microemulsion, nanomedicine, transdermal, permeation

## Abstract

To overcome the poor water solubility of total flavones of *Arisaematis rhizoma*, microemulsions (MEs) can be used as a carrier for transdermal administration to promote their solubilization and skin permeability. Here, we investigated the physical compatibility of MEs in hydrogels and their skin permeation-enhancing effects. Transparency of microemulsion-based hydrogels (MBGs) was analyzed to evaluate ME compatibility with different hydrogel matrices. Transmission electron microscopy (TEM) and Fourier transform infrared (FTIR) spectroscopy were used to explore the microstructures of MBGs and ME–hydrogel combinations. Uniform and transparent MBG was obtained by adding 1% sodium hyaluronate (SH) to the optimized ME. MBG prepared with SH as a matrix expressed pseudoplastic-fluid and shear-thinning characteristics, making it easy to apply in clinical settings. No new FTIR peak occurred in the MBG compared with ME and hydrogel matrix, indicating a physical combination of ME and the polymer network gel. Nanoscale droplets of ME migrated in the gel network, and the migration capacity and *in vitro* transdermal permeation flux negatively correlated with SH concentration in the gel system. In conclusion, in MBGs, ME can keep nanoscale droplets migrating in the hydrogel network, thereby enhancing transdermal drug delivery.

## 1. Introduction

Microemulsions (MEs) have been widely used as drug delivery systems owing to their advantages of enhanced permeation, improved drug solubility, and easy preparation (El Maghraby, 2012; Park et al., 2021; Theochari et al., 2021). However, the low viscosity of MEs makes topical administration difficult and inconvenient for patients (Heuschkel et al., 2008; Zhu et al., 2009; Scomoroscenco et al., 2021). To increase the viscosity of MEs, various kinds of gel matrix, such as carbomer, carrageenan, xanthan gum, chitosan, Poloxamer 407, and hyaluronic acid, have been added to MEs to produce microemulsion-based gels (MBGs) (Chen et al., 2006; 2013; Starychova et al., 2014; Zhang et al., 2020; Patel et al., 2021). Selecting a suitable gel matrix can improve the adhesion and spreading of MEs to the skin, block the diffusion of drugs in MBGs, and reduce the effective diffusion coefficient, thereby prolonging the action time of drugs (Chen et al., 2006; Zhao et al., 2006; Bachhav and Patravale, 2009).

The diffusion mechanism of ME droplets in gels is critical for stabilizing and enhancing ME penetration. The possible organogel structure model was first proposed based on light and X-ray scattering data, which showed that the spherical shape of ME droplets was maintained in the gel, and subsequently, MBGs were proposed to comprise an extensive, rigid, interconnected network of gelatin/water rods stabilized by a monolayer of surfactant, and coexisted with a population of conventional water-in-oil ME droplets (Jenta et al., 1997). Scan electron microscopy images at higher magnifications provide a direct evidence that the ME liquid droplets are entrapped in the gel matrix during polymerization (Gulsen and Chauhan, 2005). Transmission electron microscopy (TEM) is one of the most important techniques for studying the ME microstructures (Hajjar et al., 2018), and recently, TEM was used to investigate the microstructures of ME particles in a gel (Zhang et al., 2020). However, it is not clear whether the ME droplets can diffuse freely in the gel network.

*Arisaematis rhizoma* (AR) is a traditional Chinese medicine with analgesic, anticoagulant, anti-melanogenic, and anti-inflammatory effects (Chen et al., 2012; Kim et al., 2018). To date, AR remains an important agent for the treatment of tetanus and arthritis (Dong et al., 2015; Zhao et al., 2016). However, AR has been reported to exert several side effects with high-dose application, and conventional formulations of AR (such as cataplasm and tincture) have disadvantages of poor transdermal permeation, and seriously limit its use at an increased dosage and enhances the risk of adverse effects (Huang et al., 2011; Tomlinson et al., 2000). ME can be used as a carrier for transdermal administration of the total flavones of AR, which can promote its skin permeability (Shen et al., 2014a, 2014b). To improve the viscosity of ME, drug-loaded ME can be dispersed in a hydrogel. In this study, carbomer and sodium hyaluronate (SH) were selected as gel matrices because they are commonly used as gelling agents in hydrogels (Wan, 2015; Zhang et al., 2020; Zhu, 2020). The aim of this study was to investigate the physical characteristics of MBGs prepared with different types and concentrations of gel matrix, to elucidate the diffusion mechanism of ME in hydrogels, and reveal the *in vitro* transdermal permeation of total flavones of *Arisaematis rhizome* (TFAR)-loaded MBGs.

## 2. Materials and methods

### 2.1. Materials and animals

Apigenin (98% purity) was purchased from the Shanghai Institute for Drug Control (Shanghai, PRC). Schaftoside and isoschaftoside (98% purity) were supplied by Chengdu Mansite Pharmaceutical Co., Ltd. (Chengdu, PRC). *A. rhizoma* was obtained from Sichuan Chengdu Lotus Pond Traditional Chinese Medicine Professional Market (Chengdu, PRC). TFAR was prepared in our laboratory; it contained 0.73% ± 0.04% schaftoside, 0.21% ± 0.01% isoschaftoside (determined by HPLC), and 2.16% ± 0.06% total flavones (assayed by ultraviolet spectrophotometry). Cremophor^®^ EL was donated by BASF (Ludwigshafen, Germany). Transcutol^®^ P was provided by Gattefossé (Paris, France). SH was purchased from Shandong Freda Biotechnology Co., Ltd. (Shandong, China). Carbomer 980NF was obtained from Shanghai Yunhong Chemical Preparation Excipient Technology Co., Ltd. (Shanghai, China). All other HPLC- or analytical-grade chemicals were purchased from Sinopharm Chemical Reagent (Shanghai, China).

Sprague Dawley rats, weighing 180–220 g, were provided by the Animal Experiment Center of Shanghai University of Traditional Chinese Medicine (permit number, SYXK [Hu] 2009-0069). The rat's abdomen was depilated and then sacrificed humanely. The abdominal skin was quickly cut off, and the subcutaneous tissue was removed. The isolated skin was cleaned with normal saline and then stored at −20 °C for storage.

### 2.2. HPLC analysis method

Schaftoside and isoschaftoside were analyzed using an LC-2010A HT liquid chromatography system (Shimadzu, Kyoto, Japan) equipped with a Diamonsil^®^ C_18_ reverse-phase column (4.6 mm × 250 mm, 5 μm). The mobile phase was composed of acetonitrile (A) and 0.2% (v/v) phosphoric acid solution (B). The gradient elution program was as follows: 10–13% A for 0–10 min, 13–14% A for 10–35 min, and 14% A for 35–50 min. The flow rate was 1.0 mL·min^−1^. The column temperature was maintained at 30 °C, and the detection wavelength was set at 340 nm. The inter- and intra-day relative standard deviation values for schaftoside were 1.52% and 2.12%, respectively, and the corresponding values for isoschaftoside were 2.96% and 2.56%, respectively. Samples obtained from the experiments were filtered through a 0.45-μm filter membrane before being automatically injected into the HPLC system.

Apigenin was analyzed using an LC-2010A HT liquid chromatography system (Shimadzu) equipped with a Diamonsil^®^ C_18_ reverse-phase column (4.6 mm × 250 mm, 5 μm). The mobile phase consisted of (A) methanol and (B) water. Chromatographic separation was performed with an isocratic elution of 65% A and a flow rate of 1.0 mL·min^−1^. The column temperature was maintained at 30 °C, and the detection wavelength was set at 270 nm. The linear regression equation for apigenin was as follows: *A* = 42369C − 948.73 (*r* = 0.9999). This method was proven to be linear in the range of 0.11–111.8 µg·mL^−1^. The inter- and intra-day relative standard deviation values for apigenin were 0.56% and 1.39%, respectively. Samples from the experiments were filtered through a 0.45-μm filter membrane before being automatically injected into the HPLC system.

### 2.3. Preparation of MEs

MEs were prepared according to our previous study (Shen et al., 2014b). The drug (2%) was dissolved in the lipid excipients (mixture of ethyl oleate (4.0%), Cremophor^®^ EL (18.0%), and Transcutol^®^ P (18.0%), and purified water (58%) was added dropwise to the mixture to form MEs at 25 °C.

### 2.4. Preparation of MBGs

MBGs were obtained by adding SH or carbomer to the MEs at 25 °C after swelling for 24 h. When carbomer was used as the gel matrix, MBGs were obtained by adding an appropriate amount of carbomer and triethanolamine to the MEs.

### 2.5. Appearance and transparency of MBGs

The appearance was observed visually. The transparency of MBGs was analyzed using an ultraviolet spectrophotometer (UV-765; Shanghai Jingke Scientific Instrument, PRC) at 600 nm.

### 2.6. TEM analysis

TEM images of MBG prepared with SH, hydrogel prepared using SH, MBG prepared with carbomer, and hydrogel prepared with carbomer were acquired to investigate the morphology and structure of the formulations. A drop of diluted MBG solution was directly deposited on a copper grid; the excess was removed using a filter paper. Next, one drop of 1% phosphotungstic acid solution was placed onto the grid for 3 min to allow negative staining, and the excess fluid was removed with a filter paper. Samples without negative staining were obtained using the same procedure. After drying, the morphology was observed under a transmission electron microscope (Tecnai 12; Philips, Netherlands).

### 2.7. Fourier transform infrared spectroscopy (FTIR) analysis

FTIR was performed to examine the interaction between MEs and gel. An appropriate amount of KBr powder was used to homogenize and press the tablet with 5 g freeze-dried hydrogel. In contrast, 5 mL ME and 5 g MBG were detected directly, as their freeze-dried products were liquid and semi-solid, respectively. The FTIR spectra of MEs, hydrogels, and MBGs were obtained using an FTIR spectrophotometer (Avatar 330; Thermo Nicolet, USA).

### 2.8. Rheology analysis

The rheological behaviors of MBGs were prepared using different formulations of ME. The viscosity of the prepared MBGs was measured at 25 °C using an MCR101 rheometer (Anton Paar, Austria). The shear rate was set to increase gradually from 0.01 to 1000s^−1^, and the rheological curve between shear viscosity and shear rate was recorded. Three replicate measurements with fresh samples were performed for each test.

### 2.9. Diffusion of ME droplets in hydrogels

**Figure 1.**
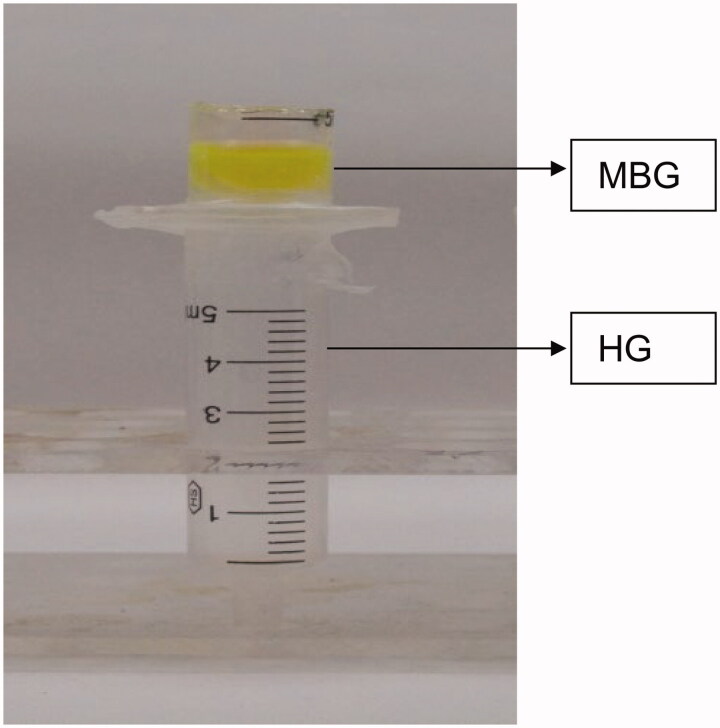
Diffusion of microemulsion droplets in hydrogel (HG).

To quantitatively analyze the permeation rate of MBGs, apigenin, a flavonoid component of AR, was used to determine the osmotic rate of MBGs in hydrogels. Apigenin content in the diffusion layer was quantitatively analyzed using HPLC. The experimental method is shown in Figure 1. MBGs were placed on the blank hydrogel for 1, 2, 4, 6, and 8 h. Changes in the hydrogel layer were also observed. After 8 h, the MBGs were removed, and apigenin in the hydrogel layer was extracted with ethanol and subsequently analyzed by HPLC.

The diffusion behaviors of MBG and a physical mixture of ME and gel matrix in hydrogel were studied. Rhodamine 110-labeled MBG was prepared by adding apigenin and rhodamine 110 to the lipid excipients (a mixture of ethyl oleate, cremophor^®^ EL, and Transcutol^®^ P), followed by dropwise addition of purified water, and subsequently, SH addition. A rhodamine 110-labeled physical mixture was obtained by directly adding a mixture of apigenin, rhodamine 110, and lipid excipients into the hydrogel. Next, 1 g of each sample was placed on the blank hydrogel and the changes in the hydrogel layer were observed.

Rhodamine 110- and apigenin-loaded MBGs with different concentrations of SH (1%, 2%, and 3%) were prepared and placed on the upper surface of the corresponding hydrogel and the changes in the hydrogel layer were observed visually.

After the MBGs diffused for a predetermined time, the hydrogel layers were investigated by TEM.

### 2.10. Transdermal delivery assay of MBG *in vitro*

A Franz diffusion cell (Tianjin Fulansi Electronic Science and Trade, Tianjin, PRC) consists of donor and receptor compartments. Each donor compartment had a surface area of 2.0 cm^2^. Excised rat skins were fixed between the two compartments. The receptor compartments were maintained at 37 ± 0.5 °C in a water bath and stirred with a magnetic bar at 300 rpm. The donor compartment was loaded with 2 g MBG. Samples were collected at fixed time intervals from the receptor compartment. The lost quantity was replaced with an equal quantity of receptor fluid, equilibrated to 37 ± 0.5 °C. The studies were performed in triplicate, and the samples were determined using HPLC.

### 2.11. Stability assay

The stability of the TFAR-loaded MBG was monitored at room temperature for 3 months. The pH values, rheological properties, as well as schaftoside and isoschaftoside concentrations of each sample were measured.

### 2.12. Statistical analysis

The results are expressed as the mean ± standard deviation. Statistical analyses were performed using Student’s *t*-tests in SPSS software (v 13.0; IBM, Co., Armonk, NY, USA). *p*-Values < 0.05 were considered statistically significant.

## 3. Results and discussion

### 3.1. Transparency

**Figure 2.**
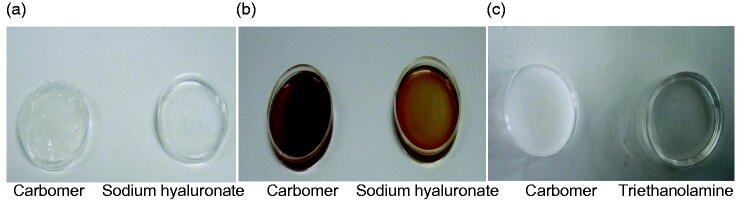
Appearance of MBGs. (a, b) MBGs prepared with different gel matrices. (c) Changes in the appearance of microemulsions after the addition of carbomer and triethanolamine.

The transparencies of ME and MBG were 87.32% ± 3.41% and 84.59% ± 0.10%, respectively, indicating the transparency of the system decreased when SH was added to ME. The appearance of MBGs prepared with SH and carbomer was significantly different (Figure 2(a–b)). When SH was added to the ME, no obvious changes were observed in clarity and transparency. In contrast, when carbomer and triethanolamine were added into the ME system, the clarity of the system decreased significantly, the system turned white opaque. This may be because carbomer is soluble in the coemulsifier, which causes the coemulsifier to lose its coemulsifying effect, resulting in a decrease in the solubilization of the oil phase, so that the oil phase overflows and demulsifies (Gulsen and Chauhan, 2005). To explore the reason for demulsifying, carbomer and triethanolamine were added to the ME, respectively. When only carbomer was added, the system became turbid whereas the triethanolamine system remained clear (Figure 2(c)). Therefore, it was speculated that the addition of a carbomer led to demulsification.

### 3.2. TEM

**Figure 3.**
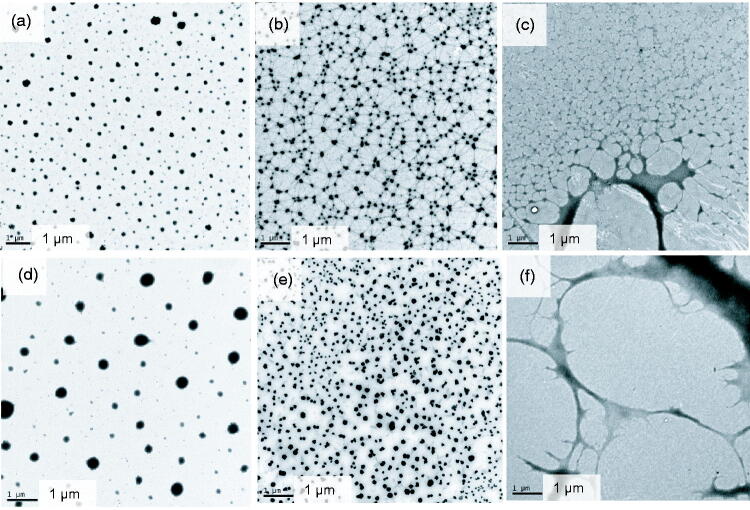
TEM images of MBGs and hydrogels. (a) MBG prepared with sodium hyaluronate with negative staining. (b) MBG prepared with sodium hyaluronate without negative staining. (c) Hydrogel prepared with sodium hyaluronate. (d) MBG prepared with carbomer with negative staining. (e) MBG prepared with carbomer without negative staining. (f) Hydrogel prepared with carbomer.

After negative staining, the morphology of ME was clearly observed, but no gel network structure was observed (Figure 3(a,d)). When SH was used as the gel matrix, the ME was arranged in a uniform emulsion droplet shape. However, when the carbomer was used as the gel matrix, the volume of the droplet increased, whereas the quantity decreased significantly. The addition of a carbomer caused ME demulsification, which was consistent with the transparency test results. When the sample was not negatively stained, ME was observed in the SH gel (Figure 3(b)). The ME was filled in the gel network structure with emulsion drops, whereas no obvious mesh structure was observed in the carbomer sample (Figure 3(e)).

### 3.3. FTIR spectroscopy

**Figure 4.**
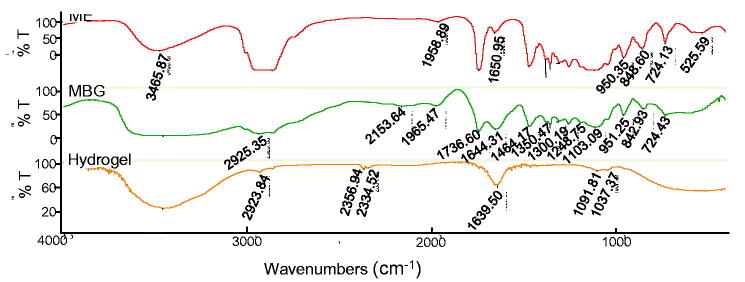
Infrared spectrogram of microemulsion, MBG, and sodium hyaluronate hydrogel.

Compared with those of hydrogel and ME, the FTIR spectrum of MBG showed no new absorption peak (Figure 4), indicating that the ME and gel were physically combined; that is, ME was distributed via ME droplets in the gel network structure.

### 3.4. Rheology

An appropriate viscosity of MBGs is important for ensuring good adhesion. It has been reported that the diffusion of drug molecules from the preparation to the skin surface can be slowed when the preparation is excessively viscous (Furubayashi et al., 2007). To identify the factors that affect the viscosity of MBGs, the rheological behavior of MBGs prepared with different types of gel matrix, different concentrations of gel matrix, and different drug loadings were studied.

**Figure 5.**
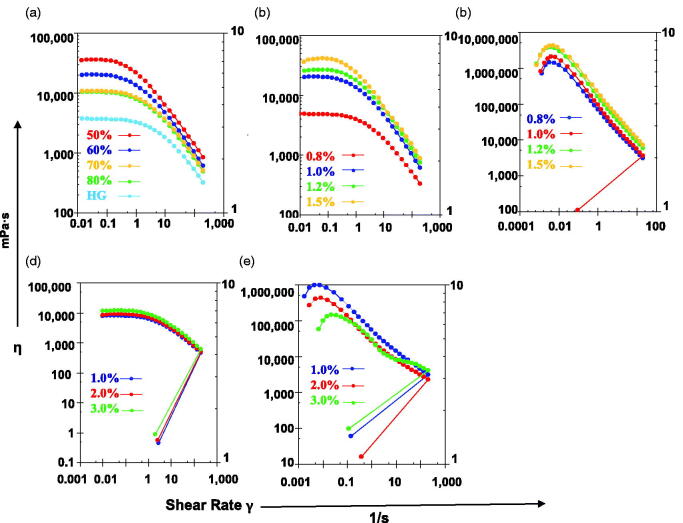
Rheological property of MBGs. (a) Rheological property of MBGs prepared with different water content. Rheological property of MBGs prepared with different concentrations of sodium hyaluronate (b) and carbomer (c). Rheological property of MBGs with different drug loading when the gel matrix used was sodium hyaluronate (d) and carbomer (e).

As shown in Figure 5(a,b,d), when the shear rate was 0.01–0.1 s^−1^, the viscosity of ME was almost unchanged, indicating that the ME had a yield value. At larger shear rates, the viscosity decreased with an increase in shear rate, indicating that the MBG was a pseudoplastic fluid. The viscosity of the pseudoplastic fluid increased at low shear rates and decreased at high shear rates. This characteristic is conducive to the storage and clinical applications of MBG.

With increasing water content, the viscosity of MBG decreased (Figure 5(a)). However, when the water content in the ME reached a certain level (70%–80%), the viscosity changed slightly. Perhaps the gel matrix had been sufficiently swollen, and the excess water formed the outer phase of the ME, filling in the mesh structure, which did not affect the porosity of the network. Furthermore, when the gel matrix and water content were the same, the viscosity of the hydrogel was lower than that of the MBG, suggesting that the water content has a significant influence on viscosity, and the MBG remained a pseudoplastic fluid.

The MBG prepared with SH and carbomer had different rheological properties. The viscosity of the MBG prepared with SH changed slightly at a low shear rate, and then decreased with an increase in shear rate. SH -formed MBG was a pseudoplastic fluid (Figure 5(b)). At low shear rates, the viscosity of the MBG prepared with carbomer increased with increasing shear rate, showing a dilatant flow, which may be due to insufficient water absorption and swelling. Subsequently, the viscosity decreased with increasing shear rate, showing a pseudo ductile flow (Figure 5(c)). The increase in gel matrix dosage may help form a tight network structure and increase the viscosity of the system (Chen et al., 2013).

Drug-loading amount had almost no effect on the viscosity of the MBG prepared with SH (Figure 5(d)). The viscosity of the MBG prepared with carbomer decreased with an increase in drug loading (Figure 5(e). The effect of drug loading on the rheological properties of MBG was related to both the drug and gel matrix. For example, when poloxamer 407 was used as the gel matrix, the viscosity of triptolide-loaded MBG was lower than that of blank MBG (Chen et al., 2013). The viscosity of indomethacin-loaded MBG increased with an increase in indomethacin concentration, indicating that the drug interacted with the polymer chains, which led to the weakening of interactions between them and, consequently, weakening of the whole gel structure. This tendency was only observed when the drug loading was 3%, and there were no significant differences when the drug loading was 1% (Froelich et al., 2017). Therefore, in the preparation of MBGs, the influence of the above factors on the viscosity of the system should be fully considered.

### 3.5. Diffusion behavior of MBGs in hydrogels

**Figure 6.**
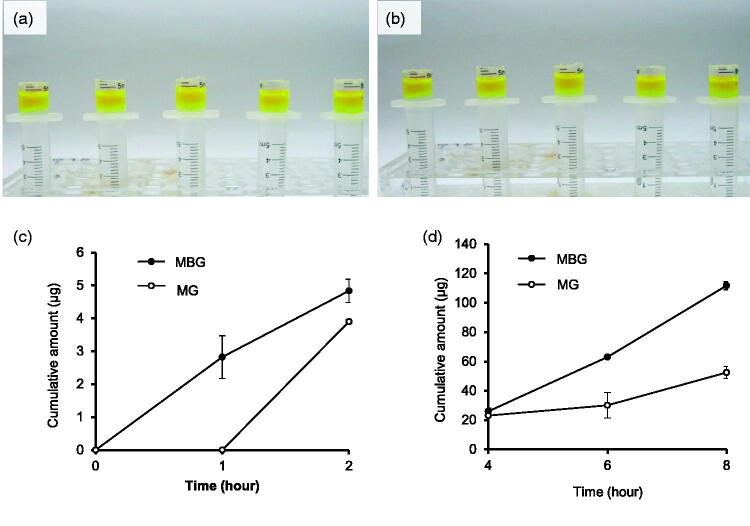
Diffusion of MBG (a) and its physical mixture gel (MG) (b). Diffusion of apigenin in MBG (c) and MG (d) (*n = 3*).

There was no significant difference in the diffusion behavior between MBG and the physical mixture gel (MG) at same viscosity (Figure 6(a and b)). However, the apigenin content in the hydrogel layer showed that the amount of the diffused drug was larger in the MBG than that in the MG (Figure 6(c–d)). This may be because the apigenin in MBG could diffuse freely with the nanoscale droplets in the gel network, whereas the apigenin in MG should first migrate from the MG to diffuse into the gel network.

**Figure 7.**
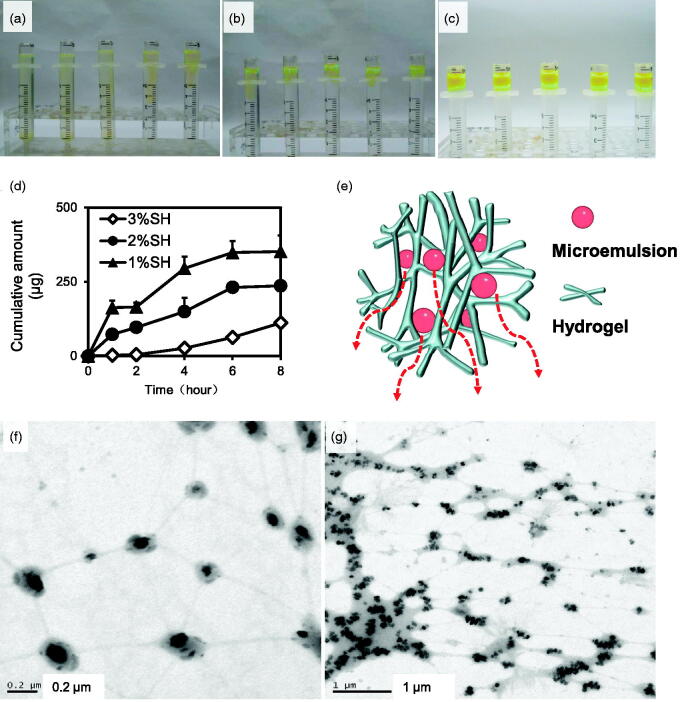
Diffusion of MBGs prepared with different concentrations of sodium hyaluronate: (a) 1% sodium hyaluronate, (b) 2% sodium hyaluronate, and (c) 3% sodium hyaluronate. Diffusion of apigine in MBGs prepared with different concentrations of sodium hyaluronate (*n* = 3) (d). Schematic diagram of microemulsion droplets diffusing through the hydrogel network (e). TEM images of hydrogel after diffusion for 8 h (f, g).

With increasing amount of SH, fluorescence diffusion became slower (Figure 7(a–c)), whereas apigenin diffusion decreased (Figure 7(d)), indicating an increase in the viscosity of the system and tightening of network structure, which was not conducive to the diffusion of ME. After MBG was diffused for 8 h, the microstructure of the hydrogel layer was observed using TEM. The morphology of the ME dispersed in the hydrogel maintained complete morphology in the gel network (Figure 7(f–g)). This finding is consistent with the proposed MBG structure based on small-angle neutron scattering (Kantaria et al., 1999) and the results of our previous study (Zhang et al., 2020). This suggests that the ME is stable in the SH-formed hydrogel, which is critical for stabilizing and achieving the penetration enhancement of ME s. Moreover, the presence of ME droplets in the gel network indicated that the ME droplets embedded in the polymeric matrix could diffuse freely (Figure 7(e)). This is similar to the results of fluorescence correlation spectroscopy, which revealed that the ME droplets are permanently placed inside the matrix and can freely diffuse in the network (Mastrangelo et al., 2017).

### 3.6. Transdermal delivery of MBGs *ex vivo*

**Figure 8.**
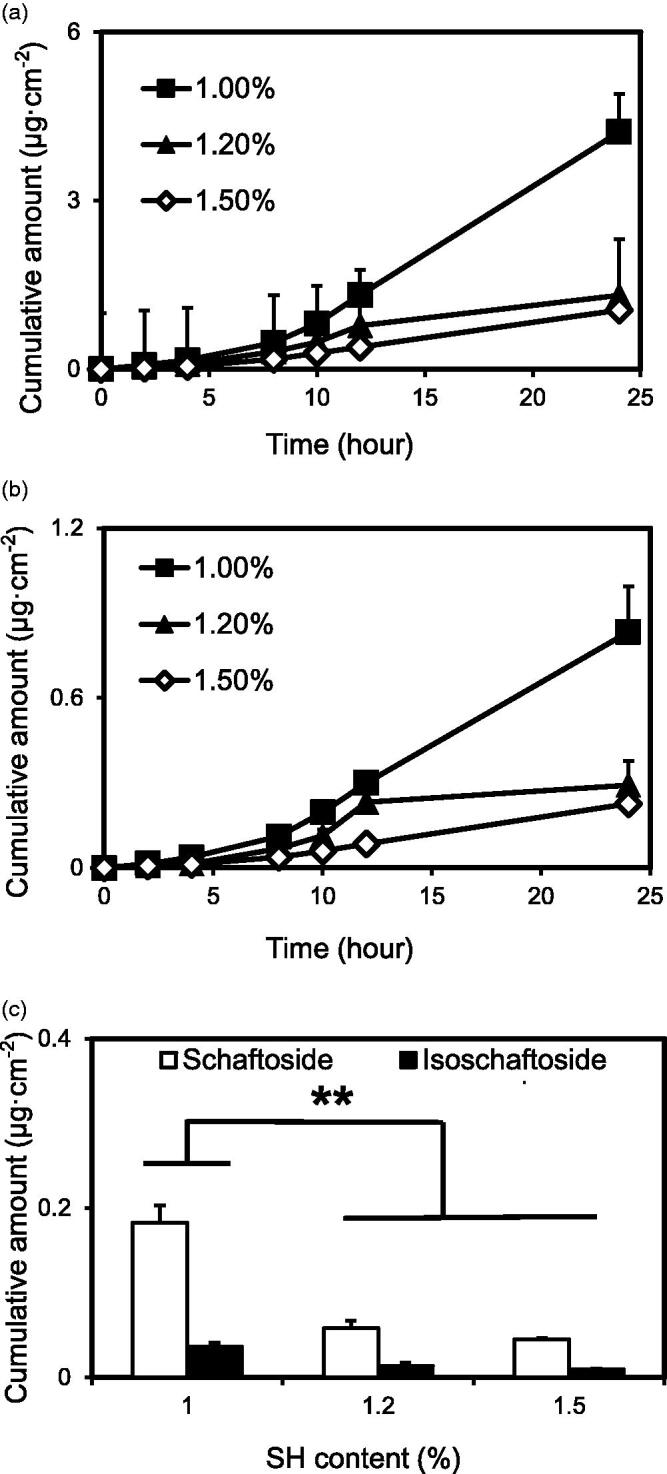
(a) *Ex vivo* skin permeation profiles of schaftoside from MBGs prepared with different concentrations of sodium hyaluronate (*n* = 3). (b) *Ex vivo* skin permeation profiles of isoschaftoside from MBGs prepared with different concentrations of sodium hyaluronate (*n* = 3). (c) *Ex vivo* skin permeation profiles of isoschaftoside from MBG prepared with different concentrations of sodium hyaluronate (*n* = 3) (compared with 1.2%, * *p* < .05; compared with 1.5%, * *p* < .05).

The cumulative amount and transdermal flux of drug versus time are presented in Figure 8(a–c). The cumulative amounts and transdermal flux of schaftoside and isoschaftoside decreased with increasing concentrations of SH. There were significant differences between the 1.0% SH group and the 1.2% and 1.5% SH groups. Owing to the increase in the gel matrix concentration, the viscosity of the system increased, whereas the penetration rate decreased (Sabale and Vora, 2012).

### 3.7. Stability

**Figure 9.**
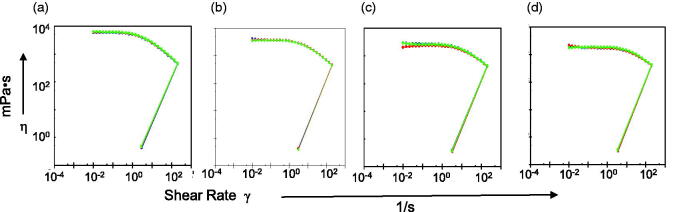
Changes in rheology with time. (a) 0 month. (b) 1 month. (c) 2 months. (d) 3 months.

TFAR-loaded MBGs showed no significant changes in appearance as well as schaftoside and isoschaftoside concentrations after 3 months of storage at room temperature. The schaftoside and isoschaftoside concentrations were stable at 94.77–96.55% and 90.00–96.47%, respectively. The pH value too remained stable at 4.70–4.80. There was no significant change in rheological behavior. The viscosity decreased slightly, but the difference was not significant (Figure 9).

## 4. Conclusions

MBGs prepared with 1% SH were in pseudoplastic fluid form with shear thinning and easily smeared characteristics. The ME and gel were physically bound in the mixture, as evidenced by the absence of new absorption peaks among ME, gel matrix, and MBG in FTIR analysis. The ME was efficiently loaded and persisted in nanoscale droplets in the gel matrix, which maintained the primary state and integrity in the network. The ME droplets diffused freely in the gel network structure, and the diffusion capacity and *ex vivo* transdermal permeation flux were negatively correlated with SH content in the gel system. In conclusion, the following mechanism is proposed for MBG action: ME migrated via nanoscale droplets in the gel network and reached the skin surface, thereby forming a concentration gradient and enhancing transdermal absorption by acting on the stratum corneum.

## Data Availability

The data presented in this study are available on request from the corresponding author.
